# Erratum to: Objective Biomarkers for Clinical Relapse in Multiple Sclerosis: A Metabolomics Approach

**DOI:** 10.1093/braincomms/fcab283

**Published:** 2021-12-08

Tianrong Yeo, Fay Probert, Megan Sealey, Luisa Saldana, Ruth Geraldes, Sebastian Höckner, Eric Schiffer, Timothy D.W. Claridge, David Leppert, Gabriele DeLuca, Jens Kuhle, Jacqueline Palace and Daniel C. Anthony. Objective Biomarkers for Clinical Relapse in Multiple Sclerosis: A Metabolomics Approach. *Brain Communications* 2021. https://doi.org/10.1093/braincomms/fcab240.

In the originally published version of this manuscript, the spelling of the co-author’s name was incorrect. The name should be “Sebastian Höckner” instead of “Sebastian Höeckner”. This error has now been corrected online.

